# Determining occupational accidents baseline ratios by considering a synthetic population: The case of Spain

**DOI:** 10.1371/journal.pone.0294707

**Published:** 2023-11-22

**Authors:** Jordi Olivella Nadal, Gema Calleja Sanz, Ignacio Fuentes Ribas, Pedro Rodriguez Mondelo

**Affiliations:** 1 Institute of Industrial and Control Engineering, and Management Department, Universitat Politècnica de Catalunya, Barcelona, Spain; 2 Management Department, Universitat Politècnica de Catalunya, Barcelona, Spain; 3 MIT Jameel Clinic, Massachusetts Institute of Technology, Cambridge, MA, United States of America; Universidad de Zaragoza, SPAIN

## Abstract

In most countries, a government agency or collaborating organization gathers information on occupational accidents. Comparisons based on a single factor such as autonomous community, activity sector or others, often leads to contradictory conclusions. The use of this information for comparison is not immediate because the different characteristics considered give place to different possible comparisons. The elaboration of a single baseline for each set of characteristics is addressed. The method proposed comes from the data available in Spain but could be applied to other cases. The method consists of: (1) selecting factors–those selected are age, sex, autonomous community and activity; (2) the generation of a synthetic population based on data from a survey and general proportions by applying the Optimal Representative Sample Weighting (*rsw*); and (3) the prediction of the accidents ratio for each set of characteristic by using a XGBoost decision trees ensemble. The results confirm the appropriateness of the method.

## 1. Introduction

Past accidents provide a strong indicator of future risk. The ultimate objective of Occupational Health and Safety (OHS) is to ensure safe and healthy working conditions by preventing injuries in a workplace. Therefore, we need to identify the risks to address and assess the importance of their possible outcomes and probability. The priorities adopted will be determined to a good extent by the analysis of the accidents occurred in the past.

More specifically, the assessment of the volume of accidents in certain industry, activity or organisation relies, to a good extent, on the details regarding the accidents both within the same context and in different scenarios. When assessing accident risk for a certain facility, the accidents occurred in this facility are the main source of information. However, often the data available is not enough to obtain significant conclusions. Even when it is, data from other facilities or workers’ groups is also useful and commonly considered. In fact, after calculating an incidence ratio of accidents, it is very common to compare it with other accident ratios. For example, assume that a company accidents ratio is 2,000 –in this paper, we consider as accidents ratio the number of accidents per 100,000 workers and year. If the industry average ratio is 1,000, this is definitely something to be worried about. These comparisons are useful to identify if the situation in an industry or a company is due to general circumstances or can be attributed to particular factors.

In most countries, a government agency or collaborating organization gathers information on accidents and provides information including aspects such as personal characteristics of the workers and the company and some details on the accident itself [1]. Based on this information, summary data and accidents ratios are usually made publicly available and can be used as baseline for comparison. However, the information provided is often descriptive and does not immediately translate to reliable comparisons, as it is described in the following example.

Let us consider the case of a young population (16–34 years), female, working in the machinery industry in a specific autonomous community and country–common characteristics to consider when analysing general accident ratios. Let us also assume that the accident ratio of this group is, for example, 4,679. How can we know if this ratio is high, low or average? We can certainly take into consideration the average of the industry, for example, but other characteristics could also be taken into account for comparison, as presented in Table 1 This example corresponds to real data of the case presented in this paper, but exact details are not included because the objective of presenting the example is only to clarify the situation that justifies this research.

**Table 1:** Example characteristics/accidents ratio.

Characteristic	Value	Average ratio
Age	16–34	3,893
Sex	Female	1,990
Autonomous community	X	2,891
Activity	Machinery	4,410
Age/sex	16-34/female	2,219

There is no clear reason to select one or the other of the averages. A possibility would be to take a value somewhere in the middle but, but it is not that straight forward. We can see, also in Table 1, that the average ratio for a narrowed group, 16-34/female value, is much closer to female average value than to 16–34 average value. Therefore, to consider an average of different values is not a reasonable solution. For a more useful comparison, we would need baselines globally reflecting the factors present in the information at a country level. Obtaining these baselines is the general objective of this paper.

### 1.1 The case of Spain

The analysis proposed will be applied to the specific case of Spain. Although the problem addressed is general, the information available depends on the specific situation and, thus, some aspects of the solution can differ. This specificity in the data available depends, firstly, on how the information is gained and stored, and, secondly, on the access of the analyst to this information.

In the case of Spain, detailed information on the accidents occurred is gathered through a centralised system of record [2]. In line with other European countries, detailed data from the accidents reports is a mandatory requirement and included in a central register [1]. Microdata of this register are made available to researchers under request to Secretaría General Técnica. Subdirección General de Estadística y Análisis Sociolaboral (estadistica@meyss.es). Additionally, detailed information on workers working at a certain moment is also gathered by public authorities through the registers on workers affiliated to the national Social Security system [3]. In this case, only a summarised version of the information is available.

### 1.2 General approach

When dealing with personal data, often data is available but it is not possible to use it due to privacy concerns. This is a very strong barrier for research on human health. The analysis of occupational accidents can be considered as one of the areas of human health research and is certainly impacted by this privacy concerns. A usual approach is the anonymisation of data, in other words, a transformation that makes it impossible to identify specific information on certain person or event. The anonymising of data can make it less detailed and, in consequence, can cause loss of information. An alternative approach is the generation of synthetic data.

Synthetic data is data that differs from reality while maintaining the same behaviour. We can consider two main types of synthetic data:

- Synthetic data prepared by someone having the real data. In this case, the real data cannot be used because of privacy concerns. However, the organisation with access can generate synthetic data with the same distribution that can be widely spread. The results of the analysis performed is expected to be the same as if obtained by using the real data.
- Synthetic data when complete real data is not available. Often the data available is only partial because it has not been registered or because it is not accessible to the researcher.

The case addressed here belongs to the second possible situation. Naturally, the method to apply to generate the synthetic data and the quality of the results depends on the partial data available. In the Spain’s case, a complementary source of information can be accessed to partly compensate the lack of detailed data on affiliated workers by generating a synthetic population. This source is EPA (Encuesta de Población Activa), a wide and nationwide survey on job situation performed systematically [4]. The microdata of this survey is publicly available. Mixing EPA data and summary data on affiliated workers will make it possible to generate a synthetic population representative of the population of workers in Spain.

The enhancement of data from a survey based on population summary data to obtain health phenomena ratios can be found in literature [5, 6]. At a more specific level, data from the EPA survey has been previously used to calculate accident ratios [7]. In these works, since the analysis was performed at an aggregated level, the generation of a synthetic population was not necessary.

By considering the data on accidents and the synthetic population of workers, we can determine occupational accidents baselines that make useful comparisons possible. To do so, we will establish the occupational accidents that we can expect under certain circumstances reflected in general statistics. This approach, using predictions to define accidents occurrence baselines, has been previously applied to road traffic accidents [8].

Technically, we will be making a prediction. However, the objective is not exactly this. In the example presented in Table 1, the features to be considered are age, sex, autonomous community, and activity. We cannot expect to predict precisely the occupational accidents ratio of a workplace only based on these parameters. Our aim is to establish an expected accidents ratio by taking into account the features considered–that is to say, we want to define baselines for comparison. The baselines should be useful both at regional, industry or company level.

The problem mentioned consists on predicting a figure and, as such, it is a regression model. In this paper, a decision-trees ensemble approach is considered. The method proposed is applied to data from Spain for the year 2019. This year has been selected because the years, 2020 and 2021, were too strongly influenced by the COVID pandemic. A regular year was considered more appropriated to check the functioning of the method proposed.

Literature on occupational accidents is wide and multifaceted [9]. Data on real accidents occurrence is one of the main ways to identify risk, and has been frequently used [10]. In particular, analyses at regional level have been addressed by considering, for example, obtaining homogeneous and comparable measures [11] and the ratios at country and activity level [12]. On the other hand, the accident occurrence in Spain at country level has also been considered, for example, to determine the factor’s influence [13], evolution over time [7], and the influence of the economic cycle [14]. However, to the best knowledge of the authors, the approach presented here is a novel contribution to literature both from the point of view of the generation of a synthetic population and for obtaining risk baselines.

The continuation of the paper is structured as following. Section 2 presents the proposed methodology, section 3 is devoted to the application of the method in the specific case of Spain, and discussion and conclusions are covered in section 4.

## 2. Problem definition and methodology

### 2.1 Problem definition

We consider a situation where the information available includes:

- Disaggregated data on occupational accidents that took place in a certain period and area, including information on some characteristics of the accident, such as the worker involved, the activity they were performing, the activity of the company and the geographical area.
- The total number of workers working in the period and area considered.
- For a list of characteristics of workers and the activity they perform, the proportion of workers in the period and area considered with each of the possible values of these features–e.g., if we consider the feature ‘industry’ we know the proportion of workers in each industry.
- A sample of the workers working in the period and area considered, with information on some demographics and the activity the workers perform. The sample was obtained from a survey.

It is possible that the data on demographics, activity and accidents of the different sources used are not exactly the same. However, we can expect some variables to be totally or partially common. The analyses performed refers to characteristics that are present in the different sources available–or can be obtained from the information they provide. Based on the information mentioned, our objective is to define baselines for comparison of occupational accidents ratios. To do so, and as an intermediary objective, a synthetic population will be generated.

### 2.2 Generation of a synthetic population

Since detailed data on workers working in the period under analysis is not available, we opt for generating a population with the same or similar characteristics–i.e., a synthetic population. To do so, in the problem defined we assume that we have a sample of the population, obtained from a survey, and the total number of workers. If we were confident that the sample reflects precisely the characteristics of the population the generation of the synthetic population would be immediate–in effect, we would divide the number of instances of the population by the number of instances of the sample and we would increase the number of instances of each specific type by this same proportion. However, most of the times samples obtained from surveys are biased because the trend to answer surveys of different groups of people is not the same.

The situation is identical to the problem addressed by raking or RIM weighting, a sample-weighting algorithm [15]. This problem corresponds to surveys with a biased sample of the population. In raking, weights are calculated for each instance in order to adjust the influence of the answers of each type of respondent to their representation in the population. Raking is considered the standard sample-weighting method [16]. Let us assume that the sample is made up of a 60% of men and a 40% of women. Let us also assume that we know that the proportion of men and women is 50%. We could adjust by multiplying the answers of each man by 0.8 (50/60) and the answers of each woman by 1.25 (50/40). The figures 0.8 and 1.2 are the weights. However, real situations are not that simple.

Let us consider a two-feature case. A simple example of this type of situation is presented in Tables 2 and 3. Table 2 shows the proportions of the variables sex and age in a population, while Table 3 shows some instances and their corresponding sex and age. A total of 8 instances of a sample are presented. In this case, the calculation of the weights is not as direct as in the case with a single instance, as we will present bellow. Now, we will explain how we propose to use the result of this problem to generate a synthetic population.

**Table 2:** Sample-weighting example: Population proportions.

Sex		Age	
Male	Female	18–45	46-
45%	55%	40%	60%

**Table 3:** Sample-weighting example: Instances and results.

Instance		Sex	Age	Weight	Percentage	Synthetic
1	Male		18–45	0.05	10%	100
2	Male		18–45	0.05		
3	Male		50-	0.116	35%	350
4	Male		50-	0.116		
5	Male		50-	0.116		
6	Female		18–45	0.3	30%	300
7	Female		50-	0.125	25%	250
8	Female		50-	0.125		

Assume that the weights presented in Table 3 (column Weight), are the result of the sample-weighting problem with this data–the sum of the proportions of the different kinds of instances verify the proportions of Table 2, as we can see in the column Percentage. If we were analysing a survey, we would multiply the answer of each person by their corresponding weight to obtain the global results. In this case, we are only interested in the composition of the population and not in weighting answers. Therefore, we will use the weights in a different way. Let us assume now that the population size is 1,000. If we multiply the sum of weights of each kind of instances by 1,000, we obtain a set of numbers of instances in the population satisfying the proportions established–as presented in Table 2, column Synthetic.

As mentioned, the problem addressed corresponds to the classical raking or RIM weighting method. However, this classical method is not fully appropriate in this case. The raking method does not give place to a single possible solution and does not take into account all the information available. To address this limitation, different solutions have been proposed [17]. Here we opt for Optimal Representative Sample Weighting (*rsw*) [18], a completely different solution method. In this method, conditions are established not only in relation to the characteristics of the population but also in relation to the weights. The objective is that the weights are as similar as possible and, thus, the characteristics of the sample are respected as much as possible.

In its most general sense, the method consists of a loss function measuring the fitting to some conditions of the population and a regularisation term, which includes condition on the weights. The equation that follows has to be minimised

$$l(f,f^{des})+\lambda r(w)$$

subject to

$$f=Fw,\;w\geq 0,\;1^{T}w=1,$$

where *f^des^* are values reflecting the characteristics of the population, *w* the weights and *F* a function transforming the weights into the values *f* to be compared with *f^des^*.

The strict Optimal Representative Sample Weighting method imposes that the proportions are fulfilled. To do so, the loss function is defined as:

$$l(f,f^{des})=\{\begin{array}{cc} 0 & f=f^{\mathrm{des}}\\ +\infty & \mathrm{otherwise} \end{array}$$

and the condition on weights can typically be the maximisation of the entropy of the weights

$$r(w)=\sum_{i=1}^{N}w_{i}\log w_{i}$$

where N is the number of elements of the sample.

In the specific case analysed here, we consider the following variables:

**Table:** 

*s*_*is*_	Categorical variables with the value of a list of characteristic *i* (*i* = 1..*I*) of the instance *s* (*s* = 1..*S*) of the sample–we assume we know the percentages per category in the population and this is only possible when variables are categorical.
*x*_*isj*_	Binary variable equal to 1 when the instance of *s* (*s* = 1..*S*) of the sample has the value *j* (*j* = 1..*J_i_*) for the characteristic *i* (*i* = 1..*I*) and 0 when it has not–the number of possible values *J_i_* depends on the characteristic.
*y*_*ks*_	Binary variables representing, for each instance of the sample *s* (*s* = 1..*S*), the values *x_ijs_* in a single sequence with *k* elements (*k* = 1..*K*), where $K=\sum_{i=I}J_{i}$ and $y_{sk}=x_{sij}\;if\;k=\sum_{i'=1..i-1}J_{i'}+j$ The data is included in a single matrix in order to adapt it to the *rsw* structure. To illustrate this formulation, let’s assume that we are considering two characteristics, Female/Male and Senior/Young, and in the population, the proportions are 40/60% and 55/45% respectively. For a set of two instances (Female/Male, Senior) and (Male, Young), the *y* matrix would be $y={\left(\begin{array}{cccc} 1 & 0 & 1 & 0\\ 0 & 1 & 0 & 1 \end{array}\right)}^{T}$, the vector of weights would be $w={\left(w_{1}\;w_{2}\right)}^{T}$, *y*.*w* would be the participation of each type of category after applying the weights and $f^{des}={\left(.4\;.6\;.55\;.45\right)}^{T}$ would be the desired values.
*p*_*k*_	Desired value of the proportion of instances, based on data on population, having, for the characteristic *i*, the value *j*, with $k=\sum_{i'=1..i-1}J_{i'}+j$
*w*_*s*_	Weight corresponding to the instance *s* (*s* = 1..*S*) of the sample

Then, we define the function *F* as the matrix:

$$F=y_{ks}$$

and the desired values as

$$f_{k}^{des}=p_{k},\;k=1..K$$

We will use the weights of the instances of the sample to generate a synthetic population. To do so, we have to define *C*, the set of possible combinations of values of the characteristics where

$$c_{il}\in C$$

is the value of the characteristic i (i = 1..I) of the possible combination l (l = 1..L), and

$$L=\prod_{i=1..I}J_{i}.$$

The variable *m_l_* (l = 1..L), which represents the number of elements to include in the synthetic population with characteristics *l*, will be calculated with the formula:

$$m_{l}=N\cdot \sum_{s=1..S;s_{si}=c_{li},\forall i,i=1..I,m=1..M}w_{s}$$

where *N* is the number of instances in the population.

### 2.3 Baseline determination

To determine the baselines, we define two additional variables,

**Table:** 

*t*_*in*_	Value of the characteristic *i* (*i = 1*..*I*) of the instance *n* (*n = 1*..*N*) of the synthetic population.
*a*_*im*_	Value of the characteristic *i* (*i = 1*..*I*) of the instance *m* (*m = 1*..*M*) of the register of accidents, where *M* is the total number of accidents in the register.

To calculate the ratio of accidents we make the following calculations–that is to say, we divide the number of accidents with values of characteristics *c_ij_* by the number of instances of the synthetic population with the same values of characteristics:

$$r_{l}=\frac{\left|\left\{m|c_{il}=a_{im},\forall i,i=1..I,m=1..M\right\}\right|}{\left|\left\{n|c_{il}=s_{in},\forall i,i=1..I,n=1..N\right\}\right|}$$

Then, we can define a prediction model with *c_il_*, the value of the characteristics, as explanatory variables, and *r_l_*, ratio of accidents, as explained variable. We will predict the ratios of accidents based on the characteristics considered. These predictions can be used as baselines with which to compare real ratios.

To perform the prediction, a method based on decision trees seems appropriate in order to capture the complex relations between the different variables that could exist. Given the potentially high number of variables and possible values, a decision tree ensemble algorithm is applied and, in particular, the XGBoost, which has been proved very efficient in predicting accidents risk based on context variables [19–21]. The appropriated parameters of the algorithm are obtained through a wide test of parameters with different training/test sets. A linear-based forecast will be performed for comparison.

Finally, the forecast for each combination of characteristics is performed by using the parameters selected and by considering the average of the results obtained from different training/test sets, to prevent a bias due to the training/test set used. The training set includes all the instances available. However, to prevent overfitting, the instance which ratio is being predicted and the most similar ones are excluded.

## 3. Application of the method to the case

### 3.1 Selection of variables and data transformation

The analysis performed takes into account three sources of information on Spanish occupational accidents and workers for the year 2019. The data, source, kind of data and number of registers are presented in Table 4. Only features present in the three sources can be used. The variables selected are age, sex, activity and autonomous community. Other features in relation to Social Security regimes and self-employment condition could also be taken into account. However, it has been judged that they depend largely on legal regulations and could give way to doubtful conclusions.

**Table 4:** Data sources.

Data	Source	Kind of data	Registers
Occupational accidents with world leave [2].	Ministerio de Trabajo y Economía Social.	Microdata provided to researchers for research purposes.	678,426
Social Security affiliated workers [3].	Ministerio de Trabajo y Economía Social.	Public summary data.	Summary
EPA, a work conditions national survey [4].	Instituto Nacional de Estadística. INEbase.	Public microdata.	655,817

The aggregation of the data is not the same in the three sources mentioned. The least aggregated measure had necessarily to be taken. In particular, the EPA uses a 10-blocks activity classification (Table 5), which is also used here. Additionally, the data on the ages of the affiliated workers is presented in blocks. For this reason, the variable reflecting ages has to be in blocks and, thus, categorical. The other variables are categorical by nature. Finally, to consider a limited number of possible values of the variables maintaining the most relevant information has been considered preferable for the application of the methods proposed. Because of this, three age blocks are considered and Autonomous Community is preferred to provinces. Finally, the age data is aggregated by dividing the most common working age range (20 to 64 years) into three blocks. Additionally, ages under 20 years and over 64 years are included in the first and third blocks obtained, respectively. The variables and blocks are showed in Table 6.

**Table 5:** Activity classification based on CNAE-2009, used by EPA.

Code	Activity
0	Agriculture, forestry and fishing (CNAE-09: 01, 02 and 03)
1	Food, textile, leather, wood and paper industry (CNAE-09: from 10 to 18)
2	Extractive industries, oil refining, chemical and pharmaceutical industries, rubber and plastics industry, electricity supply, gas, steam and air conditioning, water supply, waste management. Metallurgy (CNAE-09: from 05 to 09, from 19 to 25, 35 and from 36 to 39)
3	Manufacture of machinery, electrical equipment and transport material. Industrial installation and repair (CNAE-09 from 26 to 33)
4	Construction (CNAE-09: from 41 to 43)
5	Wholesale and retail trade and its installations and repairs. Auto repair, hospitality (CNAE-09: from 45 to 47, 55 and 56)
6	Transportation and storage. Information and communications (CNAE-09 from 49 to 53 and from 58 to 63)
7	Financial intermediation, insurance, real estate activities, professional, scientific, administrative and other services (CNAE-09: from 64 to 66, 68, from 69 to 75 and from 77 to 82)
8	Public administration, education and health activities (CNAE-09: 84, 85 and from 86 to 88)
9	Other services (CNAE-09: from 90 to 93, from 94 to 96, 97 and 99)

**Table 6:** Variables selected.

Field	Content
Age	Age blocks: less 35, 35 to 49, 50 and more.
Sex	Gender: Female, Male.
Activity	Main activity, 10-blocks classification based on CNAE-2009 and used by EPA: see Table 5.
Autonomous Community	Autonomous Community where the main activity takes place: a total of 17 Autonomous Community and 2 Autonomous cities.

The data from the EPA survey needs some transformations in order to obtain the selected data, as presented in Table 7. The field TRAREM is used to release the cases of people not working during the past week. In effect, as mentioned before, the survey includes people that are working when they are being surveyed. Additionally, the field PROEST is blank when the worker works outside Spain, and was used to release these cases. The remaining registers made counting the number of workers corresponding to each age, sex, activity and Autonomous Community block surveyed by EPA in 2019, possible.

**Table 7:** Transformation of data from EPA 2019.

Field	Content	Transformation
TRAREM	Whether they have been working the past week or not	None
EDAD5	Age in 5 years blocks	Less than 35, 35 to 49, 50 and more
SEXO1	Gender	None
ACT1	Main activity, 10-blocks classification based on CNAE-2009	None
PROEST	Province where the main activity takes place	Autonomous community or city

The CNAE-2009 activity codes are used by the Spanish Instituto Nacional de Estadística. However, until the third level of classification the CNAE-2009 classification follows NACE Rev. 2. NACE is the “statistical classification of economic activities in the European Community” and is the subject of legislation at the European Union [22]. Additionally, the two autonomous cities, Ceuta and Melilla, have been considered together due to the similar geographic characteristics.

The summary data on affiliated workers also requires some processing. The specific tables on workers affiliated to the national Social Security system considered [4] are shown in Table 8. The summary data on two variables, Age and Sex, is showed in Table 9 to illustrate the kind of information used.

**Table 8:** Tables with data on affiliation considered.

Table	Content
AFI-6	Affiliated workers by activity (Divisions) and age.
AFI-21	Affiliated workers by province, gender and age.

**Table 9:** Summary data on age and sex.

Age	Population share	Sex	Population share
<35	0.25135	M	0.53601
35–49	0.43737	F	0.46399
>49	0.31128		

On the other hand, the information on accidents also requires some modifications. The fields considered are shown in Table 10. The name and description of each filed is included, altogether with the D field number. Delta is a software system used in most of the Spanish autonomous communities to register occupational accidents data.

**Table 10:** Fields of the data on accidents considered.

Name (and Delt@ field number)	Description
Type of accident (2)	Type of accident (1 = accident, 2 = relapse)
Sex (8)	Sex (1 = men, 2 = women)
Economic activity of the center (31)	Coding according to CNAE-2009
Province (34)	Provincial code of the company
Place of the accident (46)	1 = In center or usual place; 2 = In displacement; 3 = In itinere; 4 = In another center or place
Age of the injured worker on the day of the accident	Age

The field “Type of accident” makes it possible to identify relapses, which are discarded. Additionally, following the criteria of the official Spanish reports [23], road accidents when travelling to or from the workplace (“in itinere”) are discarded. The field “Place of accident” makes that visible. Finally, the fields on economic activity, province and age need to be transformed into the common selected variables indicated in Table 6.

### 3.2 Synthetic population generation

Following the methodology proposed in subsection 2.2, a synthetic population was generated based on the data selected and appropriately transformed. The *rsw* method is implemented by using the library developed by the same authors proposing the method [24]. The hyperparameters, shown in Table 11, are standard. Although the maximum number of iterations established was 10^6^, the calculation process ended at iteration 7,800 because the value of the parameters of control were reached. The entropy of the solution is 12.204, whereas the entropy of the uniform distribution is 12.357 –thus, original values are highly respected.

**Table 11:** rsw method hyperparameters.

Losses = rsw.EqualityLoss
Regularizer = Entropy
maxiter = 100000
eps_rel = 1e-6, eps_abs = 1e-6

### 3.3 Baselines determination

The next step is the determination of baselines in accordance with the characteristics of the population considered and the ratio of accidents, which are determined by following the procedure presented in subsection 2.3. A numerical test was performed to establish the most appropriated hyperparameters. The fix and the selected parameters are shown in Table 12. A total of 100 tests with different train and test subsets were performed for each possible combination of parameters. The train and test subsets were randomly generated with a 33% testing size.

**Table 12:** XGBoost hyperparameters.

**Fix**	eta = 0.1, subsample = 0.8, objective = ’count:poisson’, colsample = 0.3
**Tested**	max_depth = [2,3,4,5,6,10]
	learning_rate = [0.05, 0.1, 0.3, 0.5]
	n_estimators = [150, 200, 250, 300, 500, 750, 1000, 1500, 2000]
	colsample_bytree = [0.1, 0.3, 0.5, 0.7, 0.9]
**Selected**	max_depth1 = 2; learning_rate1 = 0.2; n_estimators1 = 150; colsample_bytree1 = 0.7

The main criteria for selecting a set of hyperparameters was the minimum score on the test data of 10 tries. The 100 hyperparameter sets with the best minimum score have been chosen. To select one of them, a little difference between the average of accuracies of the 10 training sets and the average of accuracies of the corresponding test sets was considered. A low depth of the trees and a relatively low number of estimators have also been prioritised. All this criteria are intended to prevent overfitting. The training set average score, the test set average score and the minimum test set score were respectively 0.8425, 0.8022 and 0.7564 –the selected set is shown in Table 12.

Next, the baselines were calculated. For each instance, the training set used is the whole set, excluding the instances from the same Autonomous Community and with the same activity. If we didn’t do it this way, the forecasting could be more influenced by the values of the most similar instances than for the factors considered. Our objective here is not to predict the accidents ratio, but to know its expected value given a set of factors. The coefficient of determination of the baselines obtained in relation to the ratios was 0.7560. A linear regression was also performed for comparison purposes and the coefficient of determination was 0.6243.

## 4. Discussion of results and conclusions

The performance of the procedure proposed has been evaluated by using the scores corresponding to the specific methods used. Favourable results were obtained, as indicated in sections 3.2 and 3.3. As a result of the analysis performed, some intermediate results and an outcome, the baselines, have been obtained. A list of combinations of features has been randomly selected in order to be used as an example. The features selected, the intermediate results and the outcomes obtained are shown in Table 13.

**Table 13:** Results example (1).

Ins.	Age	Sex	Autonomous community	Activity	Number in EPA	Workers in the synthetic population	Number of accidents	Accident ratio	Baseline
(a)	35–49	M	Cataluña	Other services	221	22,455	746	3,322	2,899
(b)	>49	M	Castilla y León	Public administration, education and health activities	1,108	18,082	669	3,699	2,916
(c)	>49	M	Canarias	Manufacture of machinery, electrical equipment and transport material.	29	1,691	102	6,030	4,905
(d)	<35	F	País Vasco	Public administration, education and health activities	337	32,800	449	1,368	1,638
(e)	35–49	F	Cataluña	Construction	79	13,815	99	716	897

It is clear from these examples that, in some cases, the composition of the synthetic population is clearly different from the composition of the EPA sample. In effect, while the instance (b) of the table has 1,108 instances in EPA and 18,082 in the synthetic population, the instance (c) has 79 cases in EPA and 13,815 in the synthetic population. This is not surprising since *rsw*, the method applied, gives full priority to the fitting with the population summary data over the fitting with the sample data.

When reviewing the data results, we may be tempted to compare the accident ratios with the baselines and draw conclusions from it. Nonetheless, we think that this would not be correct. The figure on the number of accidents is obtained from real data and it is exact. The accident ratio, however, is estimated. The *rsw* method is thought to obtain the best possible fit between the synthetic population and the population itself. The conditions imposed by the method, however, do not guarantee good individual estimations, as showed in the following example.

In the example presented in Table 14, instance (a), we can see that there is a big difference between accident ratio and baseline. As already established, we cannot directly deduce anything from it. First, we notice that the baseline corresponds with what we could expect. In effect, instances with different age blocks (b and c) and autonomous communities (d) have similar baselines, but when considering same age, sex and all the activities in the same autonomous community (e), the baseline is lower. The remarkable figure here is the accident ratio. The high accident ratio can be explained because of the many accidents occurring, but also because of a bad estimation of the number of workers. The number of workers is deduced from the general information on affiliation and from the EPA poll. If the number of young women working in this autonomous community and activity is much higher than expected from the general data and very few of them answered the survey, their number can be underestimated. Conversely, the baseline reflects the information of the whole ensemble of the information available and is a trustworthy reference.

**Table 14:** Results example (&2).

Ins.	Age	Sex	Autonomous community	Activity	Number in EPA	Workers in the synthetic population	Number of accidents	Accident ratio	Baseline
(a)	E1	F	Aragón	Food, textile, leather, wood and paper industry	20	1,413	111	7,857	2,681
(b)	E2	F	Aragón	Food, textile, leather, wood and paper industry	92	5,522	145	2,625	2,387
(c)	E3	F	Aragón	Food, textile, leather, wood and paper industry	75	4,007	62	1,547	2,029
(d)	E1	F	All	Food, textile, leather, wood and paper industry	748	74,399	1,960	2,634	2,723
(e)	E1	F	Aragón	All	1,021	75,571	1,177	1,557	1,518

It has to be mentioned that the results obtained refers to sets of instances with specific values of age, sex, autonomous community and activity variables, but can be used to perform a less detailed analysis. The instance (e) from Table 14, for example, refers to workers with age in block E1, female, working in Aragon and performing any activity. The accident ratio was obtained by adding the accidents and workers corresponding to the different activities and dividing them. Additionally, the baseline was calculated in the same way by taking the numbers of accidents corresponding to the baseline of each activity.

Some limitations of this research work have to be mentioned. It has not been possible to test the homogeneity of the blocks of activity used, which correspond to the EPA survey. The availability of more detailed information would make it possible to confirm the appropriateness of the blocks or to adopt different ones. Additionally, the application of the proposed method to a single country and year could also be seen as a limitation.

Future research could overcome the two limitations mentioned. Additionally, data from several years could be analysed and the time trend influence be considered. Different criteria for aggregating data could also be considered and assessed. On the other hand, the application of the method to the analysis of the occupational accidents in other countries would require in most cases an adaptation of the model. The work could also be extended in other directions. In particular, more summary data on the population could be considered. Other factors, such as being self-employed, could also be included in the analysis. Other prediction algorithms or ensembles could be considered as well. Finally, the availability of the microdata of the population would dramatically increase the trustworthiness of the results.
